# Expression of circulating microRNA-1 and microRNA-133 in pediatric patients with tachycardia

**DOI:** 10.3892/mmr.2015.3246

**Published:** 2015-01-23

**Authors:** LING SUN, SHUO SUN, SHAOYING ZENG, YUFEN LI, WEI PAN, ZHIWEI ZHANG

**Affiliations:** 1 Departments of Pediatrics, Guangdong Academy of Medical Sciences, Guangdong General Hospital, Guangzhou, Guangdong 510080, P.R. China; 2 Departments of Cardiology, Guangdong Academy of Medical Sciences, Guangdong General Hospital, Guangzhou, Guangdong 510080, P.R. China

**Keywords:** microRNA-1, microRNA-133, markers, tachycardia, pediatric patients

## Abstract

Paroxysmal or persistent tachycardia in pediatric patients is a common disease. Certain circulating microRNAs (miRNAs) have been associated with arrhythmia. The present study investigated miRNAs in the plasma of pediatric patients with tachycardia. Forty pediatric subjects were included retrospectively: 24 with recurrent sustained tachycardia [seven cases of ventricular tachycardia (VT) and 17 cases of supraventricular tachycardia (SVT)] and 16 healthy controls. Circulating miR-1 and miR-133 in the plasma were detected by fluorescent quantitative polymerase chain reaction. miR-1 levels were significantly decreased in the arrhythmia group compared with those in the controls (P=0.004) whilst miR-133 expression levels were not significantly different between the two groups (P=0.456). Both miR-1 and miR-133 levels showed significant differences between the SVT and VT groups (P=0.004 and P=0.046, respectively), and a significant decrease in miR-1 levels was observed in the SVT group as compared with the controls (P<0.001). No significant difference was observed in the expression levels of miR-133. By contrast, miR-133 levels were significantly increased in the VT group compared with those in the controls (P=0.024), whereas no statistically significant difference was observed in the expression levels of miR-1. Receiver operating characteristic curves showed that 1/miR-1 was significant for the evaluation of tachycardia. Additionally, miR-1 produced enhanced sensitivity and specificity for the evaluation of SVT compared with miR-133, whereas miR-133 was a better marker to assess VT. This study demonstrated that miRNAs may be appropriate markers for pediatric tachycardia; miR-1 levels were decreased in the arrhythmia group compared with those in the healthy controls. Furthermore, patients with SVT had lower miR-1 expression levels while those with VT had higher miR-133 expression levels.

## Introduction

Paroxysmal or persistent tachycardia is a common condition in pediatric patients. Pediatric cardiac and non-cardiac diseases, such as anoxia, hydropenia and electrolyte imbalance, can induce arrhythmias (1). Persistent tachycardia can result in serious and potentially fatal pathologies such as heart failure; therefore, timely and effective treatment is of great clinical significance. However, the subjective symptoms of tachycardia, such as a choking sensation in the chest or palpitations, are not as evident in pediatric patients, particularly infants, as those in adult patients (2). This can lead to missed treatment opportunities and can result in severe complications, including arrhythmia, cardiomyopathy and sudden mortality. Thus, finding novel, specific biomarkers of tachycardia has great implications for the early prevention and treatment of this condition in pediatric patients and may reduce the chance of sudden mortality caused by malignant arrhythmias. At present, the treatment of arrhythmias primarily involves drug therapy and radiofrequency catheter ablation. However, this methodology is not preferred for pediatric patients since the organs of the patients are still undergoing development and vascular complications can occur (3,4). Therefore, the use of radiofrequency catheter ablation and drug therapy in the treatment of pediatric arrhythmias is limited. In recent years, clinical studies have begun attempts to control paroxysmal or persistent tachycardia in pediatric patients by gene-targeted therapy, with the aim of improving cardiac function in affected children (5,6).

microRNAs (miRNAs) are a class of single-stranded, endogenous, non-coding RNA molecules containing 20–25 nucleotides. miRNAs are formed by the miRNA-processing enzyme Dicer, from a single-stranded 70*–*90-base-pair RNA precursor with a hairpin structure. Through incomplete complementary base pairing with the 3′-untranslated region of target mRNA, miRNA can inhibit specific protein translation and expression or induce the degradation of target mRNA (7–9). miRNAs are involved in numerous key processes, including early development, cell proliferation, differentiation and apoptosis (10). To date, tissue-specific miRNAs identified in the heart have included miR-1, miR-133a/b and miR-208 (11,12). Additionally, the specific expression of circulating miRNAs has been found in various types of cancer and cardiac diseases (13). However, this study, to the best of our knowledge, is the first to report the levels of miRNAs in the plasma of pediatric patients with recurrent sustained tachycardia symptoms. Finding specific markers of tachycardia is particularly important for the early diagnosis and treatment of this disease in children.

## Materials and methods

### Blood specimen collection

This study was approved by the Ethics Committee of the Institute of Cardiovascular Diseases, Guangdong General Hospital (Guangzhou, China). The families of all pediatric patients that were included in this study signed an informed consent form. Blood specimens were collected from 40 pediatric patients between October 2012 and April 2013. The patients included 16 normal, healthy children with normal electrocardiograms (ECGs) and no history of cardiovascular disease (control group) and 24 children with recurrent sustained tachycardia who were not receiving radiofrequency ablation or antiarrhythmic drug therapy (experimental group). An ECG of the pediatric patients taken at the onset of tachycardia was used as the diagnostic criterion. Blood specimens were centrifuged within 2 h of collection (1,358 × g, 10 min, 4°C). The separated plasma and blood cells were dispensed into Eppendorf tubes and stored at –80°C prior to use.

### Primer design and synthesis

miR-1, miR-133 and U6 gene sequences were retrieved from the miRBase database (http://www.mirbase.org/) and used as a reference for designing the polymerase chain reaction (PCR) primers. The designed primers were synthesized by Shanghai Invitrogen Biotechnology Co., Ltd (Shanghai, China). The primer sequences were as follows: miR-1 forward primer, 5′-ACACTCCAGCTGGGTGGAATGTAAAGAAGT-3′ and reverse primer, 5′-TCAACTGGTGTCGTGGAGTCGGCAATTCTTGAGCAGCTGGT-3′; miR-133 forward primer, 5′-ACACTCCAGCTGGGTTTGGTCCCCTTCAAC-3′ and reverse primer, 5′-CTCAACTGGTGTCGTGGAGTCGGC AATTCAGTTGAGCAGCTGGT-3′; reverse universal primer URP, 5′-TGGTGTCGTGGAGTCG-3′; internal reference U6 forward primer, 5′-CTCGCTTCGGCAGCACA-3′ and U6 reverse primer, 5′-AACGCTTCACGAATTTGCGT-3′. The downstream primers of miR-1, miR-133 and U6 were mixed in equal volumes to obtain a concentration of 10 *μ*M for each primer.

### Reverse transcription (RT) and fluorescent quantitative PCR (qPCR)

Total RNA was extracted from the plasma using TRIzol^®^ LS reagent (Invitrogen Life Technologies, Carlsbad, CA, USA) according to the manufacturer’s instructions. The total RNA was subjected to reverse transcription (RT) by PCR immediately subsequent to extraction. The 20-*μ*l RT-PCR reaction system (ReverTra Ace-α-Reverse Transcription kit; Toboyo Co., Ltd., Osaka, Japan) contained 2 *μ*l 10X buffer, 1 *μ*l 2.5 mM deoxynucleotide triphosphate, 3 *μ*l mixed downstream primer, 0.5 *μ*l reverse transcriptase Moloney murine leukemia virus and 13.5 *μ*l RNA template. The RT-PCR program comprised 25°C for 15 min followed by 50°C for 50 min. A total of 2 *μ*l RT product (cDNA) was utilized for qPCR. The 20-*μ*l reaction systems for the qPCR detection of miR-1, miR-133 and U6 contained 10 *μ*l SYBR Premix Taq II (2X; Takara Bio, Inc., Shiga, Japan), 0.2 *μ*l 30 pmol/*μ*l upstream primer, 0.2 *μ*l 30 pmol/*μ*l downstream primer, 7.6 *μ*l dH_2_O and 2 *μ*l cDNA. The fluorescent qPCR program comprised 95°C for 5 min, followed by 40 cycles of 94–80°C (94°C for 10 sec, 55°C for 20 sec, 72°C for 10 sec and 80°C for 35 sec) using an ABI 7500 fluorescent quantitative PCR machine in plate read mode (Applied Biosystems Life Technologies, Foster City, CA, USA), which identifies genotypes, detects gene locus mutations and analyzes single nucleotide polymorpyhsms. The program ended with the preparation of a melting curve at 60–95°C.

### Statistical analysis

The standard curves of miR-1 and miR-133 were used to calculate the absolute quantities of miR-1 and miR-133. The levels of circulating miR-1 and miR-133 in the plasma are expressed as the mean ± standard deviation. Measurement data were analyzed by the independent two-sample t-test with P<0.05 considered statistically significant. The sensitivity of miR-1 and miR-133 to detect arrhythmia was tested with receiver operating characteristic (ROC) curves. All data were processed using SPSS 16.0 (SPSS Inc., Chicago, IL, USA) statistical software.

## Results

### Baseline data

This study included 40 pediatric patients: 24 with arrhythmia and 16 healthy controls (24 males and 16 females) with average ages of 6.6±3.9 and 9.8±1.8 years in the arrhythmia and control groups, respectively. In the arrhythmia group, there were seven cases of ventricular tachycardia and 17 cases of supraventricular tachycardia (SVT). Baseline data of the pediatric patients are shown in Table I.

### Circulating miRNA levels in the plasma of pediatric patients

**Figure 1:**
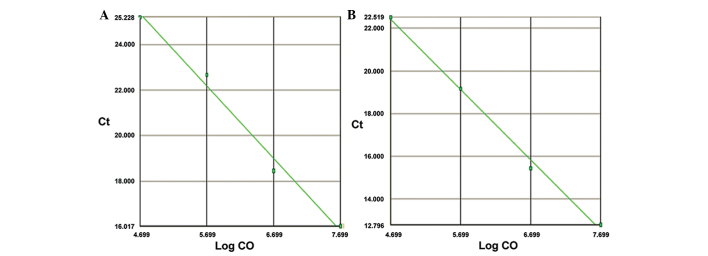
Standard curves for (A) miR-1 and (B) miR-133. miR-1, *y*=–3.186442+40.348274*x* (R^2^=0.988156); miR-133, *y*=–3.289124+37.668317*x* (R^2^=0.995739). miR, microRNA; Ct, cycle threshold; CO, initial copies.

**Figure 2:**
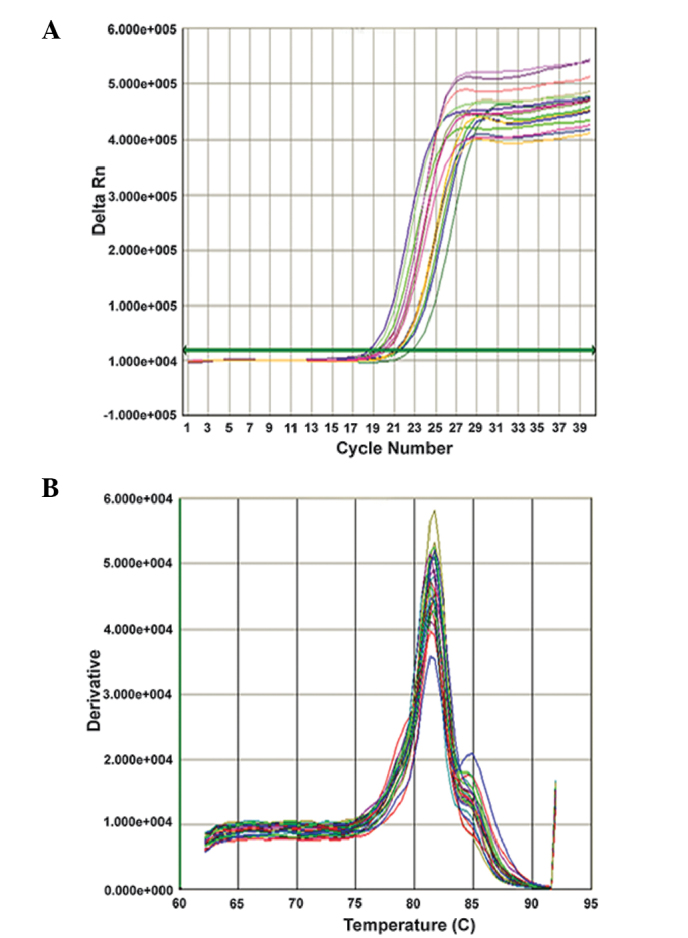
microRNA-1 (A) amplification and (B) melting curves from SYBR quantitative polymerase chain reaction detection.

**Figure 3:**
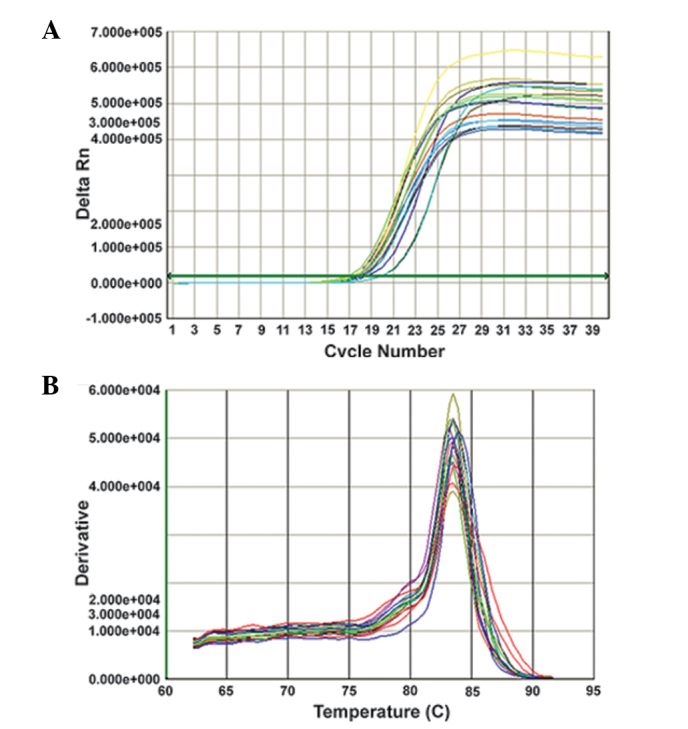
microRNA-133 (A) amplification and (B) melting curves from SYBR quantitative polymerase chain reaction detection.

The standard curves prepared by double dilution of miR-1 and miR-133 standards (*y*, cycle threshold value; *x*, Log inital copies; CO) are shown in Fig. 1. The amplification and melting curves for the qPCR are shown in Figs. 2 and 3.

**Figure 4:**
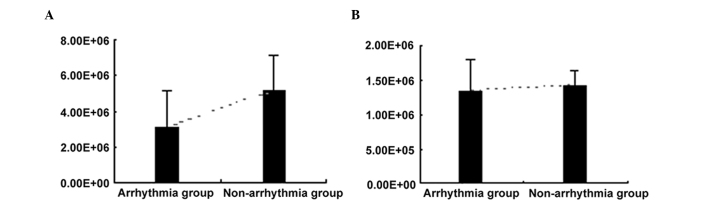
(A) miR-1 and (B) miR-133 levels in the plasma of pediatric patients. miR, microRNA.

The miR-1 levels in the plasma of pediatric patients showed a significant difference between the arrhythmia and non-arrhythmia groups (3.09×10^6^±2.11×10^6^ vs. 5.16×10^6^±1.99×10^6^ copies/ml, P=0.004), whereas no statistically significant differences were observed in the miR-133 levels between the two groups (1.34×10^6^±4.74×10^5^ vs. 1. 43×10^6^±2.03×10^5^ copies/ml, P=0.456) (Table II). The above results indicate that miR-1 levels were decreased in the plasma of the patients with arrhythmia whereas miR-133 levels exhibited no significant variation (Fig. 4).

### miRNA levels in the plasma of pediatric patients of different genders

**Figure 5:**
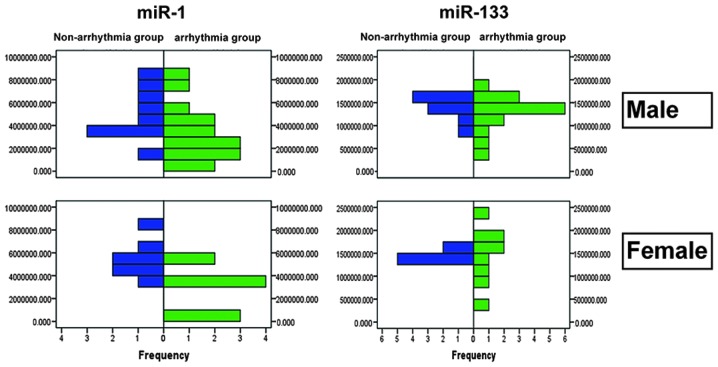
Frequency distribution of miR-1 and miR-133 in male and female pediatric patients. miR, microRNA.

The levels of miR-1 and miR-133 in the plasma of pediatric patients with arrhythmia showed no statistically significant differences between the males and females (miR-1, 3.86×10^6^± 2. 41×10 ^6^ vs. 4. 01×10 ^6^±2.15×10^6^ copies/ml, P=0.842; miR-133, 1.33×10^6^±3.47×10^5^ vs. 1.45×10^6^±4.44×10^5^ copies/ml, P=0.351) (Table III). The frequency distribution of miR-1 and miR-133 in the plasma of male and female pediatric patients with arrhythmia and controls is shown in Fig. 5.

### Sensitivity and specificity of 1/miRNA for the evaluation of arrhythmia (ROC curve)

**Figure 6:**
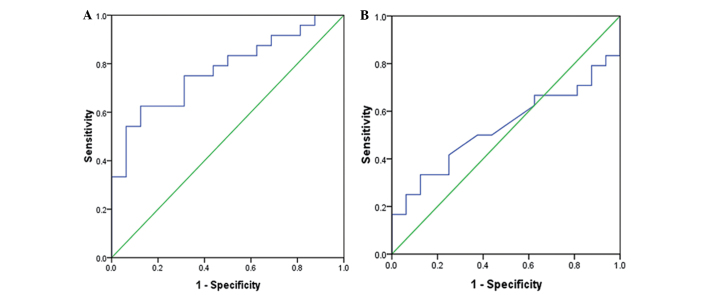
Receiver operating characteristic curve sensitivity and specificity of (A) 1/miR-1 and (B) 1/miR-133 for the evaluation of arrhythmia. miR, microRNA.

ROC curves were prepared with 1/miR-1 and 1/miR-133. Arrhythmia was defined as the positive event (Fig. 6). The results showed that the diagnosis of tachycardia with 1/miR-1 had statistical significance (P=0.004; area under the ROC curve, 0.773; 95% confidence interval of the area, 0.630–0.917), whereas the diagnosis of tachycardia with 1/miR-133 had no statistical significance (P=0.73) (Table IV).

### Subgroup analysis of miRNA levels in the plasma of patients with arrhythmia

Subgroup analysis was performed on the pediatric patients with arrhythmia for the comparison of plasma miR-1 and miR-133 levels between the SVT and VT groups (Table V). Statistically significant differences were observed in the miR-1 and miR-133 levels in the plasma between the SVT and VT groups (miR-1, 2.41×10^6^±1.62×10^6^ vs. 4.76×10^6^±2.36×10^6^ copies/ml, P=0.004; miR-133, 1.22×10^6^±5.08×10^5^ vs. 1.64×10^6^±1.69×10^5^ copies/ml, P=0.046). In addition, statistically significant differences in plasma miR-1 levels were observed between the patients with SVT and the control group (2.41×10^6^±1.62×10^6^ vs. 5.16×10^6^±1.99×10^6^ copies/ml, P<0.001) (Table VI), whereas the plasma miR-133 levels showed no significant difference between these two groups. The expression levels of miR-133 in the plasma were, however, significantly different between the VT and normal control groups (1.64×10^6^±1.69×10^5^ vs. 1.43×10^6^±2.03×10^5^ copies/ml, P=0.024) (Table VII), whereas no significant difference was observed in the plasma miR-1 levels between these two groups. Together, these results indicate that circulating miR-1 levels in the plasma of pediatric patients with SVT were downregulated, whereas miR-133 levels in the plasma of pediatric patients with VT were upregulated.

### Sensitivity of ROC curves for the evaluation of SVT and VT

**Figure 7:**
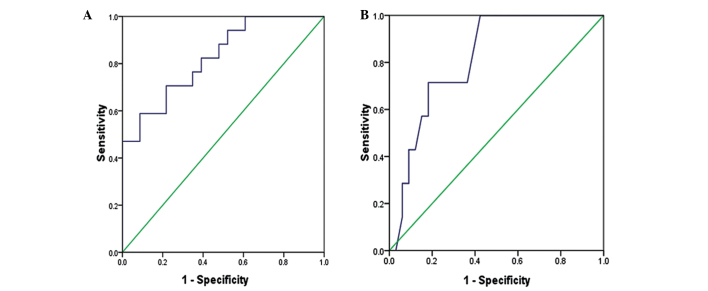
(A) Sensitivity and specificity of miR-1 for the evaluation of supraventricular tachycardia. (B) Sensitivity and specificity of miR-133 for the evaluation of ventricular tachycardia. miR, microRNA.

According to the ROC curves, miR-1 had enhanced sensitivity and specificity for the evaluation of SVT (P<0.001; area under the ROC curve, 0.826; 95% confidence interval of the area, 0.699–0.953) (Fig. 7A), whereas miR-133 had better sensitivity and specificity for the evaluation of VT (P=0.01; area under the ROC curve, 0.814; 95% confidence interval of the area, 0.671–0.957) (Fig. 7B).

## Discussion

miRNAs regulate cell proliferation and differentiation by mRNA-specific base pairing and their expression exhibits cell or tissue specificity (14–16). Studies have demonstrated that numerous miRNAs are involved in the reconstruction of ion channels by regulating gene expression in cardiomyocytes during the process of arrhythmia (17–19). At present, studies on the association between miRNA and arrhythmia are primarily focusing on pathological and physiological processes such as myocardial ischemia and cardiac hypertrophy (20–23). The interactions of miRNAs with ion channel-encoding genes and calmodulin regulate cardiac contractility, rhythm and excitement, thereby inducing arrhythmia (24,25). However, less research has been undertaken into arrhythmia in pediatric patients without organ disease, and, to the best of our knowledge, no reports are available on the specific expression of circulating miRNA in pediatric patients with persistent tachycardia.

In the present study, miR-1 and miR-133 levels in the plasma of pediatric patients with tachycardia and healthy controls were quantitatively detected by a qPCR. The results showed that circulating miR-1 levels in the plasma were lower in the patients with arrhythmia than those in the healthy controls, whilst no significant differences were observed in the miR-133 expression levels. Of note, the results of the subgroup analysis revealed that there were significant differences in circulating miR-1 and miR-133 levels in the plasma of pediatric patients between the SVT and VT groups. Additionally, circulating plasma miR-1 levels were decreased in patients with SVT, whereas plasma miR-133 levels were significantly increased in patients with VT, as compared with those in the normal controls. Together, these results demonstrate that different circulating miRNAs in the plasma of pediatric patients with SVT and VT may cause the reconstruction of various ion channels, thereby inducing arrhythmias of different types.

Myocardial ischemia-induced arrhythmia is a major cardiovascular pathology associated with miR-1 and miR-133. It has been reported that miR-1 expression is significantly increased in patients with myocardial ischemia and infarction and is causative of arrhythmias. A possible mechanism for this is that the ischemia-related abnormally high expression of miR-1 leads to a reduction in gap junction α-1 protein/Connexin43 expression and a delay in cardiac conduction, as well as to a reduction in potassium inwardly-rectifying channel, subfamily J, member 2 expression, a decline in inward rectifier current density and resting potential rise, ultimately leading to the occurrence of ischemic arrhythmia (26–29). Zhang *et al* (30) found in an animal model of myocardial ischemia that miR-1 overexpression in the ventricular heart muscle caused atrioventricular block; the underlying mechanism was associated with a reduction in Connexin43 expression and a decline in L-type Ca^2+^ currents. In the present study, none of the pediatric patients included had organic heart disease. The finding of down-regulated miR-1 expression in the plasma of pediatric patients contrasted with upregulated miR-1 expression following myocardial ischemia, indicating that pediatric SVT involves a different pathogenesis. Connexin43 is a protein component of gap junctions, involved in regulating synchronized contraction of the heart. The expression level of Connexin43 is relatively low in the sinoatrial and atrioventricular nodes, whereas its expression in the surrounding tissues is high (31). Therefore, data from the present study suggest that the pathogenesis of SVT in pediatric patients without heart disease is associated with the presence of considerable Connexin43 expression adjacent to the atrioventricular nodes and in the atrioventricular accessory pathways. The upregulation of Connexin43 expression may inhibit the expression of miR-1, leading to increases in the myocardial conduction velocity. A major limitation of the present study lies in the difficulty of obtaining myocardial tissues from pediatric patients to quantitatively determine Connexin43 expression in the myocardial tissue.

miR-133 is another miRNA with specific expression in myocardial tissue that is known to affect the autorhythmicity of heart tissue through pacemaker channels [potassium/sodium hyperpolarization-activated cyclic nucleotide-gated ion channel 2 (HCN2) and HCN4] (32,33) or potassium ion channels [potassium voltage-gated channel, KQT-like subfamily, member 1 (KCNQ1) and potassium voltage-gated channel, Isk-related family, member 1 (KCNE1)] (34,35). In miR-133a transgenic mice, miR-133a inhibits the rapid delayed rectifier potassium current encoded by the congenital long QT2 syndrome gene and the slow delayed rectifier potassium current encoded by the KCNQ1 and KCNE1 genes. Damage to the repolarizing current channels leads to the extension of action potential duration or prolongation of the QT interval (36). In the present study, miR-133 levels were increased in pediatric patients with VT; this finding may be associated with damage to the repolarizing current channels, although further investigations are required to validate this hypothesis.

This study investigated whether the levels of circulating miR-1 and miR-133 in the plasma of pediatric patients differed between male and female patients; the results showed no significant differences in miR-1 and miR-133 levels between the genders (P>0.05). However, Stauffer *et al* (37) found that gender was associated with significantly different Connexin43 and miR-1 expression, with miR-1 expression in the plasma lower in female than in male patients (37). This inconsistency between the studies may be due to the small sample size and different ethnicities and ages of patients included. Studies with a larger sample size are required, as well as comparisons between the levels of circulating miRNAs in the plasma of pediatric and adult patients.

Since differences in the levels of specific miRNAs have been identified in the plasma of pediatric patients with arrhythmias, miRNA interference technology may be used as a suitable treatment of refractory arrhythmias. To rectify the downregulation of miR-1 expression in pediatric patients with SVT, we speculate that synthetic miR-1 may be introduced into cells by exogenous, double-stranded miRNA or miRNA mimic technologies. To target the upregulation of miR-133 expression in pediatric patients with VT, miRNA antisense oligonucleotide technology and miRNA barrier oligonucleotide technology can be used to inhibit the expression of miR-133, further controlling the occurrence of arrhythmia. However, these and other relevant technologies are still in the early stages of testing.

The identification of specific miRNAs has provided a novel perspective for studying the pathogenesis of arrhythmia. Accordingly, new ideas have been considered for the future development of novel, secure and effective drugs for treating arrhythmia. The expression of specific circulating miRNA in plasma has great medical significance for early arrhythmia prevention. In the near future, gene-targeted therapy and the prevention of arrhythmia in pediatric patients are likely to have great benefit.

## Figures and Tables

**Table I:** Baseline data of the pediatric patients.

	Arrhythmic	Non-arrhythmic
Number	24	16
Gender (n male/n female)	15/9	9/7
Age (years)	6.6±3.9	9.8±1.8

**Table II:** miR-1 and miR-133 levels in the plasma of pediatric patients.

miR type	Arrhythmic (copies/ml)	Non-arrhythmic (copies/ml)	P-value
miR-1	3.09×10^6^±2.11×10^6^	5.16×10^6^±1.99×10^6^	0.004^a^
miR-133	1.34×10^6^±4.74×10^5^	1.43×10^6^±2.03×10^5^	0.456

a Indicates statistical significance. miR, microRNA.

**Table III:** miR-1 and miR-133 levels in the plasma of male and female pediatric patients.

miR type	Male (copies/ml)	Female (copies/ml)	P-value
miR-1	3.86×10^6^±2.41×10^6^	4.01×10^6^±2.15×10^6^	0.842
miR-133	1.33×10^6^±3.47×10^5^	1.45×10^6^±4.44×10^5^	0.351

N=40 patients; male, n=24; female, n=16. miR, microRNA.

**Table IV:** Sensitivity and specificity of 1/miRNA for the evaluation of arrhythmia.

1/miR type	Area under ROC curve	P-value	95% CI
1/miR-1	0.773	0.004^a^	0.630–0.917
1/miR-133	0.533	0.73	0.534–0.711

a Indicates statistical significance. miR, microRNA; ROC, receiver operating characteristic; CI, confidence intervals.

**Table V:** Comparison of miR-1 and miR-133 between the supraventricular and ventricular tachycardia groups.

miR type	Supraventricular tachycardia (copies/ml)	Ventricular tachycardia (copies/ml)	P-value
miR-1	2.41×10^6^±1.62×10^6^	4.76×10^6^±2.36×10^6^	0.004^a^
miR-133	1.22×10^6^±5.08×10^5^	1.64×10^6^±1.69×10^5^	0.046^a^

a Indicates statistical significance. miR, microRNA.

**Table VI:** Comparison of miR-1 and miR-133 between the supraventricular tachycardia and normal control groups.

miR type	Supraventricular tachycardia (copies/ml)	Normal control (copies/ml)	P-value
miR-1	2.41×10^6^±1.62×10^6^	5.16×10^6^±1.99×10^6^	<0.001^a^
miR-133	1.22×10^6^±5.08×10^5^	1.43×10^6^±2.03×10^5^	0.143

a Indicates statitstical significance. miR, microRNA.

**Table VII:** Comparison of miR-1 and miR-133 between the ventricular tachycardia and normal control groups.

miR type	Ventricular tachycardia (copies/ml)	Normal control (copies/ml)	P-value
miR-1	4.76×10^6^±2.36×10^6^	5.16×10^6^±1.99×10^6^	0.68
miR-133	1.64×10^6^ ±1.69×10^5^	1.43×10^6^±2.03×10^5^	0.024^a^

a Indicates statistical significance. miR, microRNA.
